# Rare Record of Albinism in a New Zealand Fur Seal ( *Arctocephalus forsteri* ) Pup With Observations Through the Weaning Period

**DOI:** 10.1002/ece3.72510

**Published:** 2025-11-12

**Authors:** Alasdair A. Hall, Robyn A. Grant, Jody Suzanne Weir

**Affiliations:** ^1^ Hall Marine Consulting Wellington New Zealand; ^2^ Department of Natural Sciences Manchester Metropolitan University Manchester UK; ^3^ Department of Conservation New Zealand

**Keywords:** albinism, *Arctocephalus forsteri*, colour morph, otariid, pinniped

## Abstract

The rarity of heritable pigment anomalies in marine mammals makes it challenging to assess their fitness ramifications. Potential negative effects include reduced visual acuity, greater predation risk, increased disease vulnerability, lower fertility and decreased heat absorption. Logging instances of heritable pigment anomalies can improve understandings of their impacts and potentially permit their use in understanding metapopulation dynamics. We report on a rare observation of albinism in a male New Zealand fur seal/kekeno ( *Arctocephalus forsteri* ) pup. The pup was discovered near Kaikōura during colony monitoring in February 2025. He had honey‐coloured natal pelage, bluish‐white pupils, white/translucent whiskers and pink skin on his flippers, nose, ears and around his eyes. He appeared to experience reduced visual acuity. When observed on September 1, 2025, the pup lacked muscle tone, potentially a result of his eyesight precluding typical muscle development behaviours. His survival chances postweaning may be reduced by his poor eyesight, although a previous record of albinism in a subadult conspecific demonstrates postweaning survival is possible. While abnormally pigmented individuals from other Arctocephalus species have typically been sighted at remote colonies where year‐round observations are not possible, this birth in central mainland New Zealand provides opportunities for longitudinal tracking.

## Graphical abstract

This article provides the first known scientific record of albinism in a New Zealand fur seal pup. We describe the individual with a particular focus on its sensory abilities. This individual's birth at a central mainland colony may permit continued study throughout his life, unlike many records of similar conditions in other species.

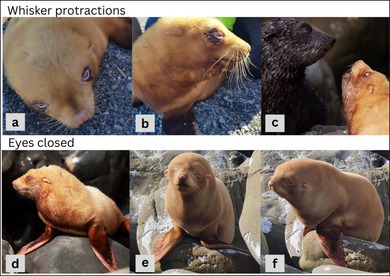

## 1 Introduction

Colour of the hair, skin and eyes in mammals is controlled by the amount of melanin production (Lamoreux et al. 2010; Lin and Fisher 2007), and anomalies in the typical colour for a given species can result from heritable genetic mutations (Shihlomule et al. 2024). In mammals, genetic mutations that can induce anomalous pigmentation are classified into albinism, leucism, melanism and piebald, each of which differs in its impacts (Acevedo and Aguayo 2008; Shihlomule et al. 2024). Unlike leucism, melanism and piebald, albinism impacts pigmentation of the eyes (as well as the nails, hair and skin) (Fertl and Rose 2009; Shihlomule et al. 2024). The fitness implications of pigment anomalies are uncertain, partly because of the rarity of their occurrence (Acevedo and Aguayo 2008; Anderson et al. 2025). Potential negative effects include reduced visual acuity (Shihlomule et al. 2024), greater vulnerability to predation, increased disease vulnerability, lower fertility and decreased heat absorption (Acevedo and Aguayo 2008; Anderson et al. 2025; Fertl and Rose 2009; Hain and Leatherwood 1982). Study of pigment anomalies is not only important for understanding the impacts on individuals, but also for their heritability means they can provide insights into meta‐population structure. For example, the spread of cream‐coloured Antarctic fur seals ( *Arctocephalus gazella* ) has been used to demonstrate the importance of South Georgia as a source population for this species post‐sealing (Hoffman et al. 2018).

Among marine mammals, atypical pigmentation has been observed in both cetaceans (Estupiñán‐Montaño and Baron 2025; Funasaka et al. 2015; Keener et al. 2008; Methion and López 2019; Nascimento et al. 2008; Pirotta et al. 2023) and pinnipeds. In pinnipeds, such anomalies have most often been reported in otariids, and particularly among Antarctic fur seals (Acevedo, Torres, and Aguayo‐Lobo 2009; Bonner 1968; De Bruyn et al. 2007; Hofmeyr et al. 2005; Romero and Tirira 2017). Recently, ocular albinism has been reported in sub‐Antarctic fur seals ( *Arctocephalus tropicalis* ) (Shihlomule et al. 2024), as has atypical lanugo coat pigmentation (du Toit et al. 2019; Jones et al. 2019). Other otariids in which pigmentation anomalies have been observed include northern fur seals ( *Callorhinus ursinus* ) (King 1983; NOAA 2020), South American sea lions ( *Otaria flavescens* ) (Acevedo and Aguayo 2008), California sea lions ( *Zalophus californianus* ) (Bartholomew and Hubbs 1952) and Steller sea lions ( *Eumetopias jubatus* ) (King 1983). Records among phocids are rarer and include Weddell seals ( *Leptonychotes weddellii* ) (Acevedo, Aguayo‐Lobo, and Torres 2009; Anderson et al. 2025), southern elephant seals ( *Mirounga leonina* ) (Bester et al. 2008), harbour seals ( *Phoca vitulina* ) (Osinga et al. 2010) and grey seals ( *Halichoerus grypus* ) (Osinga et al. 2010).

In New Zealand, New Zealand fur seal/kekeno ( *Arctocephalus forsteri* ) monitoring occurs during the species' austral summer breeding season, with surveys mostly undertaken between mid‐January and late February (Boren et al. 2006; Chilvers 2021; Hall et al. 2025). Typically, New Zealand fur seals are born with a black natal pelage and whiskers, brown eyes and dark skin. Pups moult into their adult pelage at around 3–5 months old (Taylor 1992). As adults, males typically vary from dark grey to brown in colour, while adult females vary between metallic grey and brown (Goldsworthy et al. 1997).

The only known record of albinism in New Zealand fur seals comes from the popular press (Dangerfield 2011). A New Zealand Department of Conservation (DOC) ranger reported seeing an albino subadult in 2011, which was very similar in colour to the individual recorded here (Figure 2). The 2011 sighting may have been the same individual observed as a pup four years earlier in the same location. While the location itself was not provided, the description of ‘north of Kaikōura’ likely refers to Ōhau Point.

## 2 Methods

**FIGURE 1:**
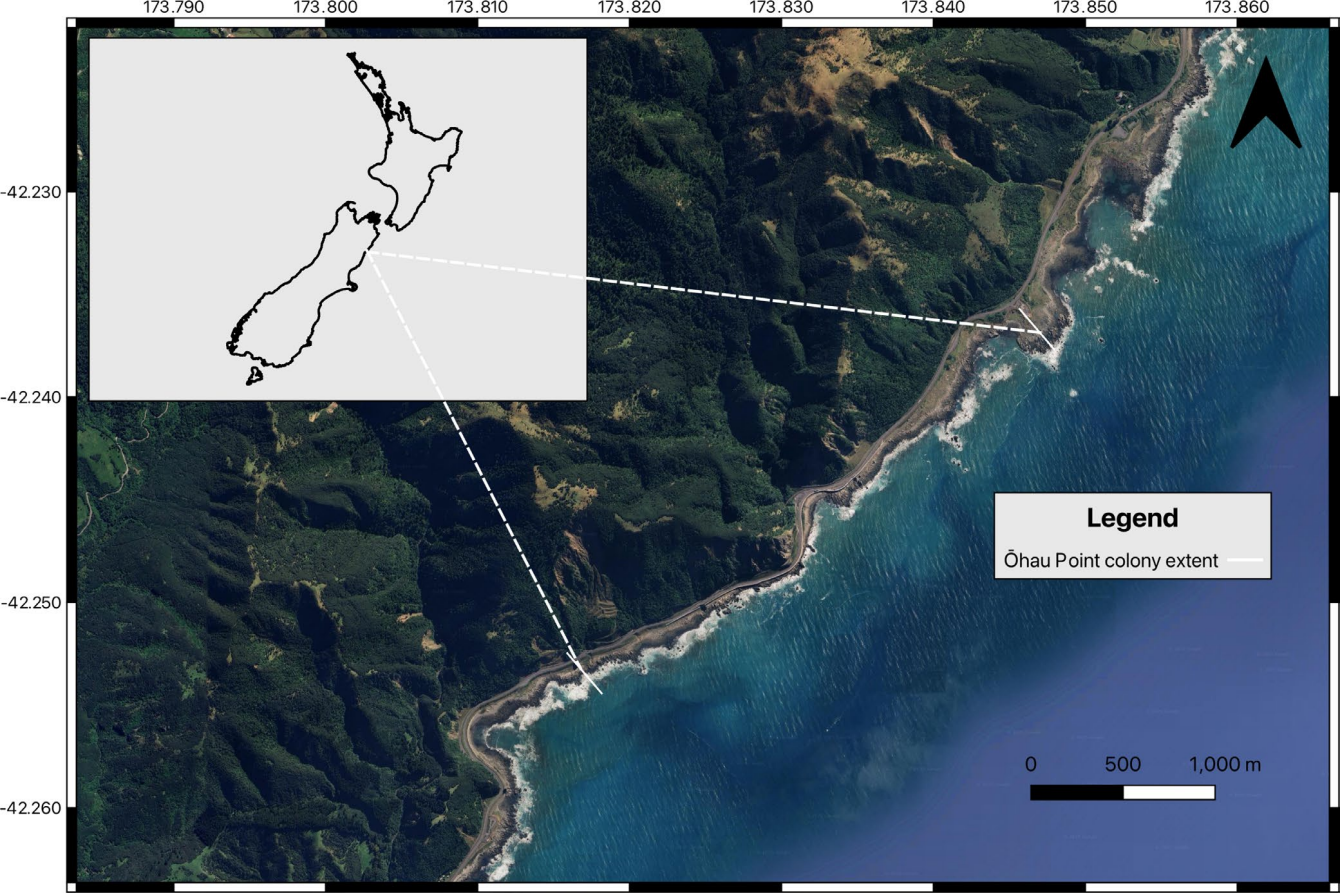
Map showing the location and extent of the Ōhau Point New Zealand fur seal colony in 2025.

Ōhau Point is on the north‐eastern coast of New Zealand's South Island, near Kaikōura (Figure 1). Annual New Zealand fur seal breeding surveys have occurred there since 2022/2023, following a gap due to impeded access after the 2016 Kaikōura earthquake (Hall et al. 2023). In the 2024/2025 breeding season, an estimated 3137 (± 87 SE) pups were produced at Ōhau Point (Hall et al., in preparation). During this survey, an albino New Zealand fur seal pup was discovered on February 15, 2025.

**FIGURE 2:**
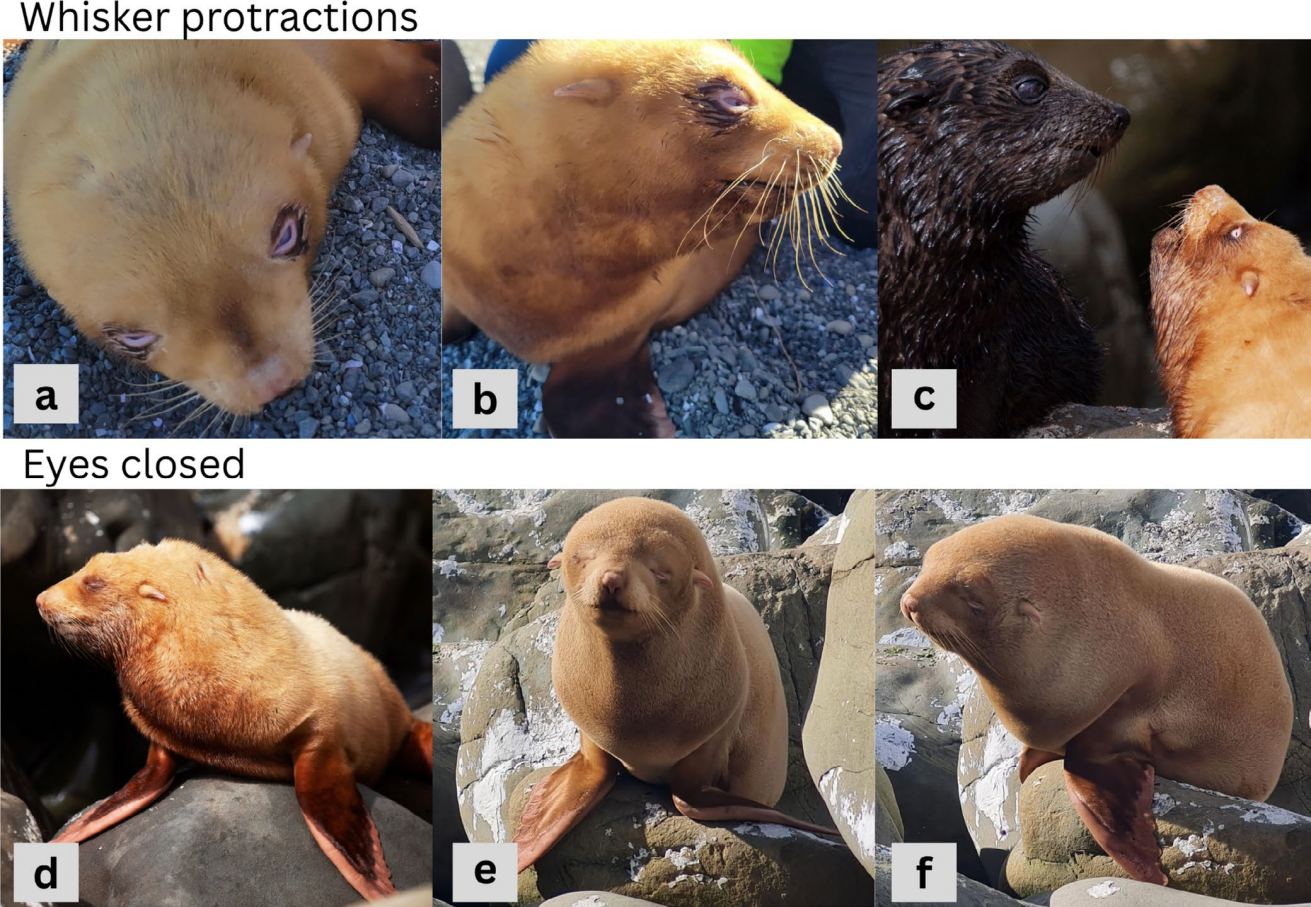
Photos of the albino pup found at Ōhau Point. Photos (a) and (b) were taken on February 15, (c) and (d) were taken on February 25th and (e) and (f) were taken on September 1st, 2025.

Upon initial discovery, the albino pup was captured, sexed and photographs taken. Given that most pupping occurred at the end of November 2024, we estimate the albino pup to have been approximately 2–3 months old at this time. He was observed again on February 17 and 25 and further photographs were taken. On all the occasions, the pup was found among other New Zealand fur seals, and on February 25 he was observed play fighting with another pup (Figure 2c). When observed on September 1, 2025, the pup was again found among other conspecifics but mostly remained stationary. Between February and September, reports from members of the public suggest he remained in the vicinity of Ōhau Point throughout the weaning period. The pup was observed by JSW throughout September and October, always in the same area of the colony, with the latest sighting at the time of writing being November 10, 2025. On October 20, the albino pup was observed suckling from his mother, who was of normal colour.

## 3 Results

The Ōhau Point albino was identified as male and had honey‐coloured natal pelage, and pink skin on his flippers, around his eyes and on his ears and nose. The pup's pupils were bluish‐white, and his whiskers were white/translucent (Figure 2a–c). He appeared to be in good body condition when initially encountered.

During all observations throughout September and October, the albino pup (aged 9/10 months) was within 100 m of where he was first discovered. Despite having undergone the juvenile–adult moult, his pelage was similar in colour to that when first discovered. While not emaciated, he appeared to lack the muscle and tone of his peers from the 2024/2025 cohort (Figure 2f). The pup appeared to experience reduced visual acuity. While surrounding individuals fled as researchers approached (as expected [Mattlin 1978]), the albino did not indicate awareness of researchers. Rather, he seemed to be alerted by the sounds of other fur seals moving over nearby rocks and moved towards those sounds.

## 4 Discussion

This report provides the first scientific record of albinism in a New Zealand fur seal. The colour of the Ōhau Point individual bears a striking resemblance to the photos of an albino northern fur seal pup discovered on the island of Tyuleny, Russia in 2020 (NOAA 2020). The observation of a ‘pale golden’ Antarctic fur seal pup on Bird Island, South Georgia from 1933 (Rayner & Etheridge 1933 in [Bonner 1968]), could also describe a similarly coloured individual. The retention of abnormal pelage pigmentation post‐moult contrasts with reports of abnormally pigmented sub‐Antarctic fur seal pups which, post‐moult, adopted more typical pelage colour (du Toit et al. 2019; Shihlomule et al. 2024). There are, however, examples of pinniped pigmentation anomalies persisting into adulthood (Acevedo and Aguayo 2008; Jones et al. 2019), including in the subadult New Zealand fur seal observed in 2011.

In addition to non‐responsiveness to human presence, the light colour of the eyes suggests that the Ōhau Point pup experiences reduced visual acuity, which has been reported previously in albino animals (Grønskov et al. 2007; Shihlomule et al. 2024). The pup's eyes were often closed (Figure 2d–f), perhaps due to not being used, or as a response to bright sunlight as pigment absence in the eyes increases photosensitivity (van Grow 2006). The pup has been observed with his eyes open, notably in the morning when the sun was less bright.

Mammalian whiskers may be able to compensate for deficient vision, and evidence suggests that blind phocid seals can forage using their whiskers (Dehnhardt et al. 1998; Adachi et al. 2022). Visual inputs can even influence the development of whisker shape in mammals; for instance, congenitally blind cats and mice have larger whiskers and larger brain areas than intact animals (Rauschecker et al. 1992). While the whiskers were pale in colour, they appeared to be a similar length and thickness to those of other pups. In February, the pup's whiskers appeared to be more curved at the tips (Figure 2b) compared to other pups, although they were much straighter in September (Figure 2e,f). The whiskers were clearly mobile, and the pup used them to guide behaviours, such as locomotion, grooming and social interactions, similar to other individuals (Figure 2c). The lack of muscle tone observed in September may be due to poor eyesight limiting the pup's engagement in muscle development behaviours such as play‐fighting and diving development (Arnold and Trillmich 1985; Gentry 1974; Spence‐Bailey et al. 2007).

It has been suggested that the lack of visual recaptures of abnormally pigmented pinnipeds may indicate poor survival chances (Acevedo, Torres, and Aguayo‐Lobo 2009). However, the observation of an albino subadult New Zealand fur seal near Kaikōura in 2011 shows postweaning survival is possible. Unlike many previous records of pigmentation abnormalities in southern hemisphere pinnipeds, which have largely occurred in remote Antarctic and sub‐Antarctic regions (Acevedo, Aguayo‐Lobo, and Torres 2009; Acevedo, Torres, and Aguayo‐Lobo 2009; Anderson et al. 2025; du Toit et al. 2019), Ōhau Point's location means the albino pup can be relocated throughout the species' long weaning period, giving researchers the chance to make novel observations of this rare individual. Once weaned, male New Zealand fur seals are likely less philopatric than females (Stirling 1971), meaning he may not return to Ōhau Point. However, if he survives, the albino pup may be easier to track through life, given his birth at a centrally located colony on New Zealand's mainland, compared to individuals born in more remote regions. Given the rarity of albinism in New Zealand fur seals, additional marking such as flipper‐tagging is deemed unnecessary at this stage.

If continued resights of this individual are possible, future research could focus on understanding how this individual's whiskers and other sensory organs are used to compensate for his poor eyesight. Additionally, as albino animals may struggle to thermoregulate as effectively as typical conspecifics (Anderson et al. 2025; Fertl and Rose 2009), there may be opportunities to compare thermoregulatory behaviour between this individual and his conspecifics to improve understandings of thermoregulatory pathways in the species.

## Author Contributions

**Alasdair A. Hall:** conceptualization (equal), investigation (equal), methodology (equal), visualization (equal), writing – original draft (lead), writing – review and editing (equal). **Robyn A. Grant:** conceptualization (equal), investigation (equal), methodology (equal), visualization (equal), writing – review and editing (equal). **Jody Suzanne Weir:** conceptualization (equal), data curation (equal), investigation (equal), methodology (equal), project administration (equal), supervision (equal), writing – review and editing (equal).

## Conflicts of Interest

The authors declare no conflicts of interest.

## Data Availability

There are no accompanying data associated with this manuscript.
